# Supplementary material for: Historical museum collections and contemporary population studies implicate roads and introduced predatory bullfrogs in the decline of western pond turtles
Source: PeerJ. 2020 Jun 12;8:e9248. doi: 10.7717/peerj.9248 (PMC7295021; doi:10.7717/peerj.9248)
Supplement: Supplemental Information 2 [file peerj-08-9248-s002.docx]

| **County** | **Species** |
| --- | --- |
| Alpine | *Emys marmorata* |
| Amador | *Emys marmorata* |
| Butte | *Emys marmorata* |
| Calaveras | *Emys marmorata* |
| Colusa | *Emys marmorata* |
| Del Norte | *Emys marmorata* |
| El Dorado | *Emys marmorata* |
| Fresno | *Emys marmorata* |
| Glenn | *Emys marmorata* |
| Humboldt | *Emys marmorata* |
| Inyo | *Emys marmorata* |
| Kern | *Emys marmorata* |
| Kings | *Emys marmorata* |
| Lake | *Emys marmorata* |
| Lassen | *Emys marmorata* |
| Madera | *Emys marmorata* |
| Marin | *Emys marmorata* |
| Mariposa | *Emys marmorata* |
| Mendocino | *Emys marmorata* |
| Merced | *Emys marmorata* |
| Modoc | *Emys marmorata* |
| Napa | *Emys marmorata* |
| Nevada | *Emys marmorata* |
| Oregon and Washington | *Emys marmorata* |
| Placer | *Emys marmorata* |
| Plumas | *Emys marmorata* |
| Sacramento | *Emys marmorata* |
| San Joaquin | *Emys marmorata* |
| Shasta | *Emys marmorata* |
| Sierra | *Emys marmorata* |
| Siskiyou | *Emys marmorata* |
| Solano | *Emys marmorata* |
| Sonoma | *Emys marmorata* |
| Stanislaus | *Emys marmorata* |
| Sutter | *Emys marmorata* |
| Tehama | *Emys marmorata* |
| Trinity | *Emys marmorata* |
| Tulare | *Emys marmorata* |
| Tuolumne | *Emys marmorata* |
| Yolo | *Emys marmorata* |
| Yuba | *Emys marmorata* |
| Alameda | *Emys pallida* |
| Contra Costa | *Emys pallida* |
| Los Angeles | *Emys pallida* |
| Monterey | *Emys pallida* |
| Orange | *Emys pallida* |
| Riverside | *Emys pallida* |
| San Benito | *Emys pallida* |
| San Bernardino | *Emys pallida* |
| San Diego | *Emys pallida* |
| San Francisco | *Emys pallida* |
| San Luis Obispo | *Emys pallida* |
| San Mateo | *Emys pallida* |
| Santa Barbara | *Emys pallida* |
| Santa Clara | *Emys pallida* |
| Santa Cruz | *Emys pallida* |
| Ventura | *Emys pallida* |
