# Supplementary figures and images for: Historical museum collections and contemporary population studies implicate roads and introduced predatory bullfrogs in the decline of western pond turtles
Source: PeerJ. 2020 Jun 12;8:e9248. doi: 10.7717/peerj.9248 (PMC7295021; doi:10.7717/peerj.9248)

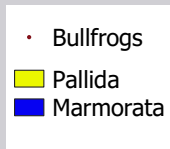

Supplement: Supplemental Information 3 [file peerj-08-9248-s003.pdf]

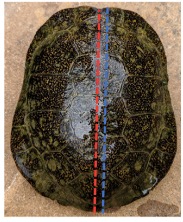

Supplement: Supplemental Information 4 [file peerj-08-9248-s004.jpg]

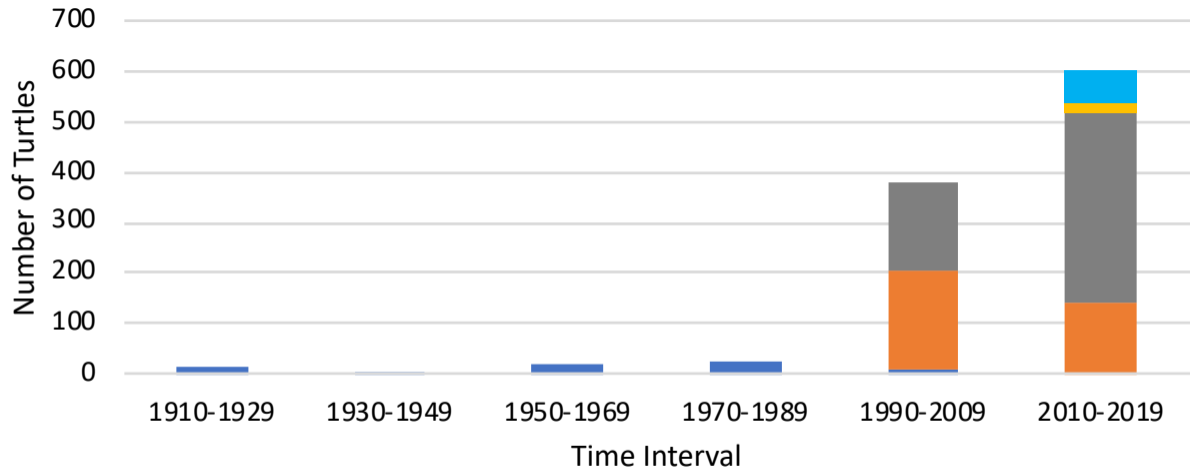

Museums Santa Monica Mountains USGS Dangermond Lake Elizabeth

Supplement: Supplemental Information 5 [file peerj-08-9248-s005.pdf]

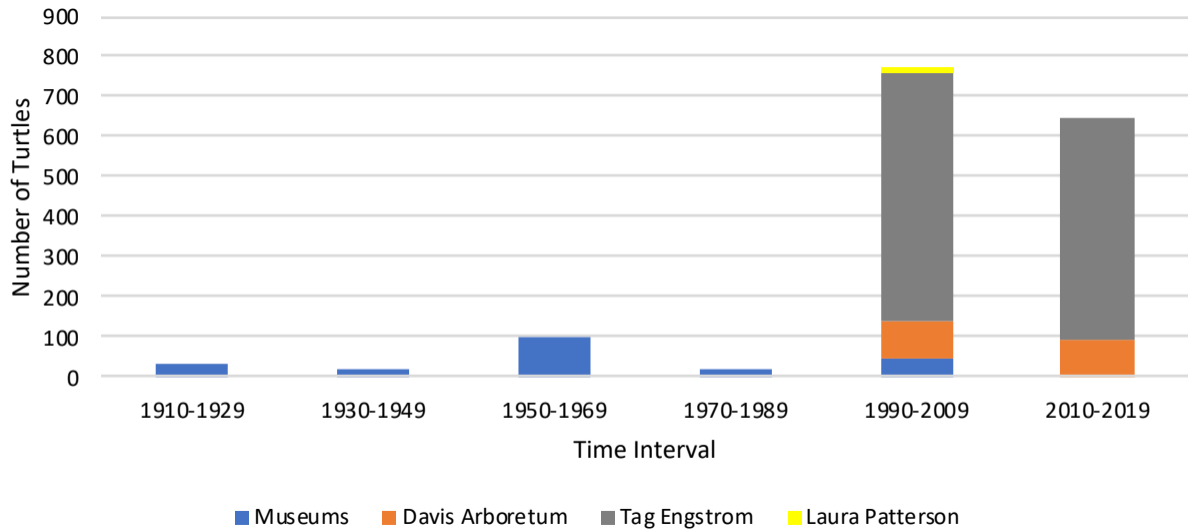

Supplement: Supplemental Information 6 [file peerj-08-9248-s006.pdf]

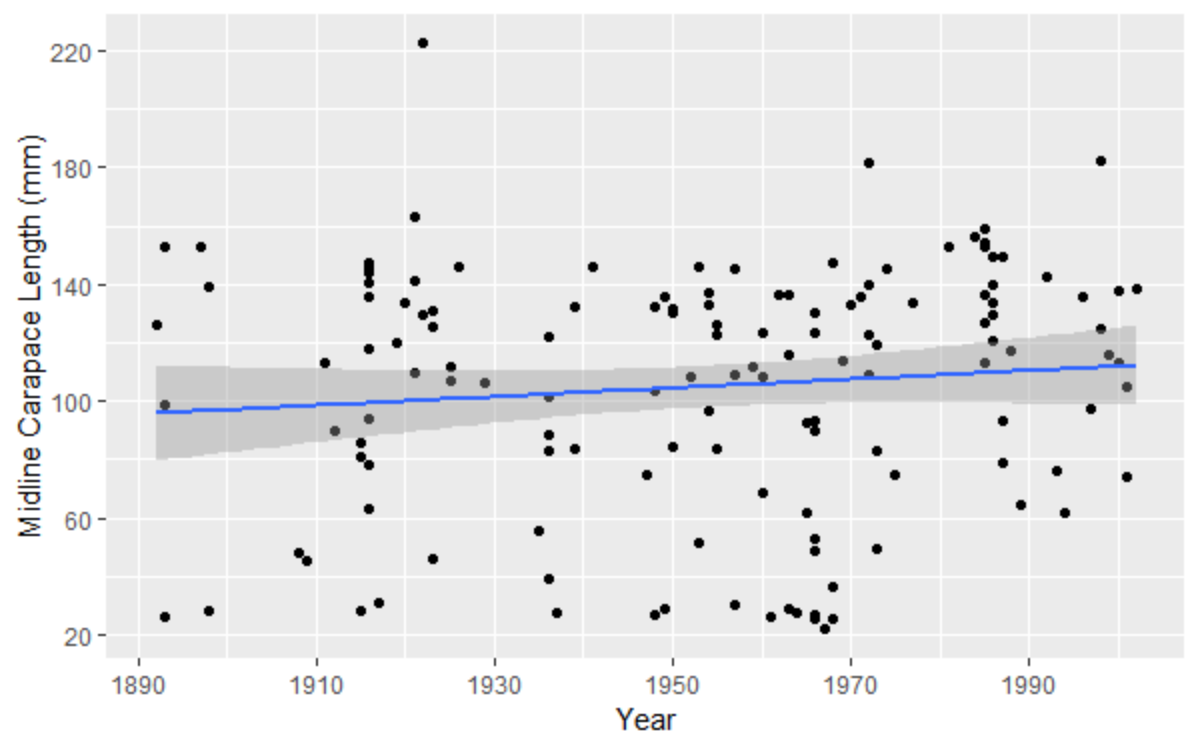

Supplement: Supplemental Information 7 — The blue trend line and grey shading account for the 95% confidence interval. [file peerj-08-9248-s007.pdf]

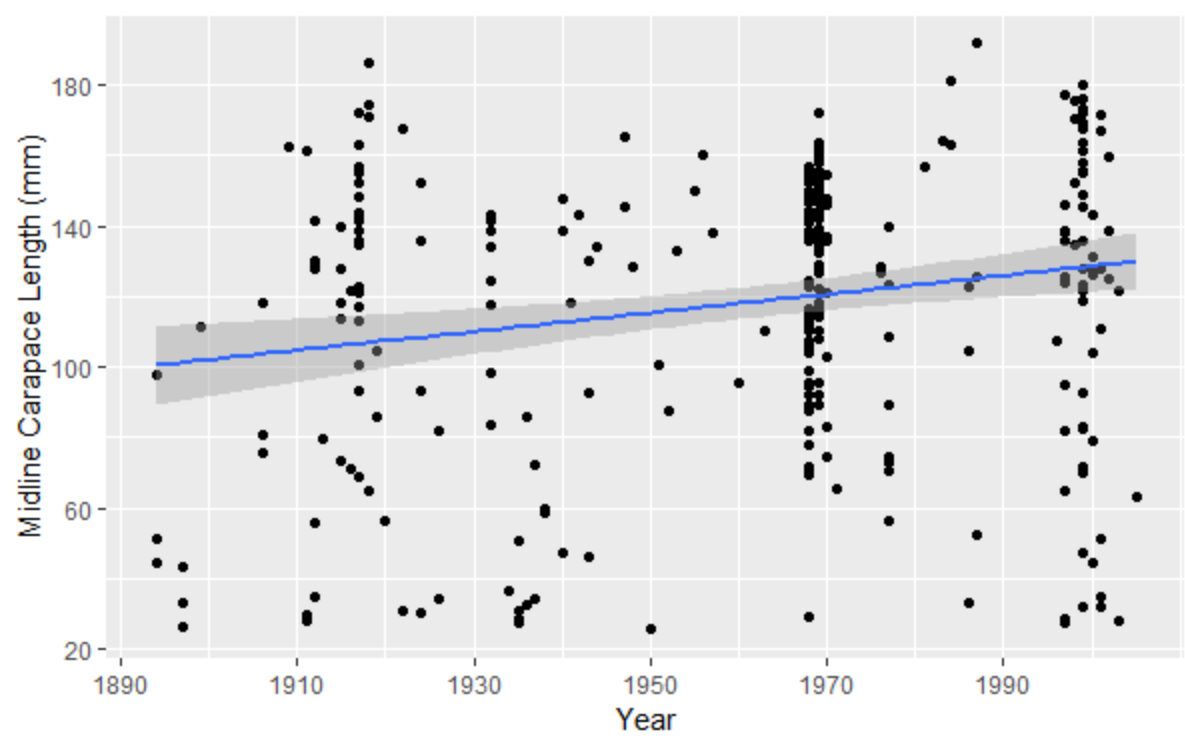

Supplement: Supplemental Information 8 — The blue trend line and grey shading account for the 95% confidence interval. [file peerj-08-9248-s008.pdf]

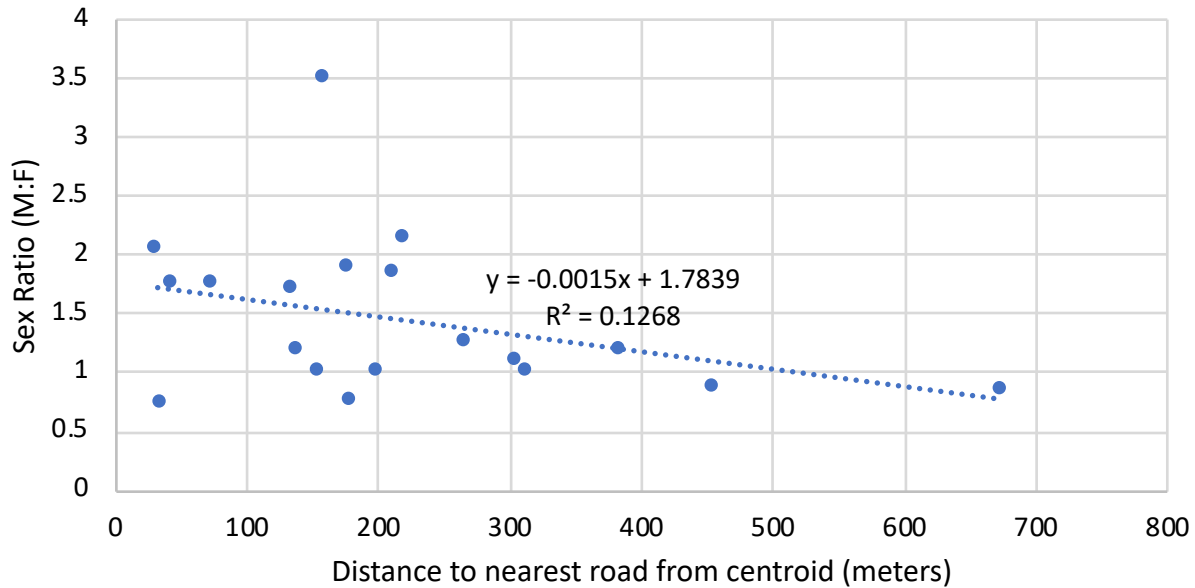

Supplement: Supplemental Information 9 [file peerj-08-9248-s009.pdf]

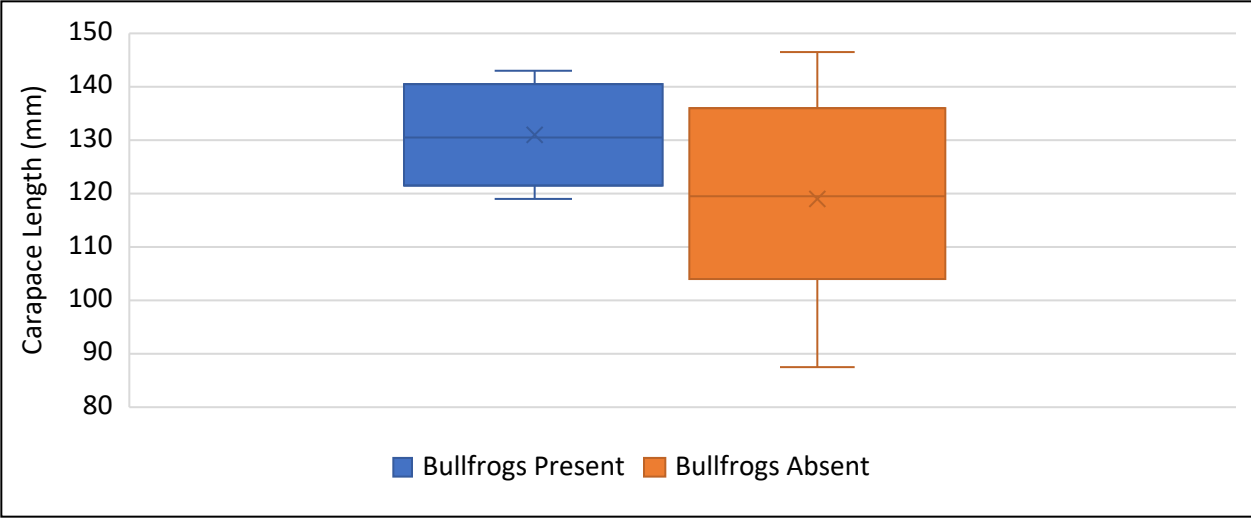

Supplement: Supplemental Information 11 [file peerj-08-9248-s011.pdf]

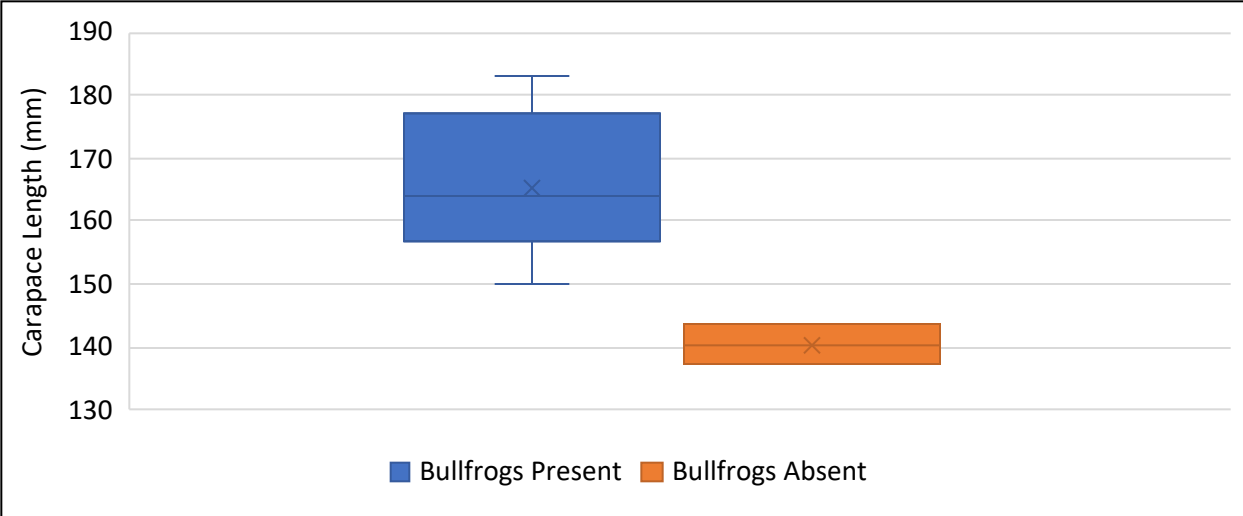

Supplement: Supplemental Information 12 [file peerj-08-9248-s012.pdf]
