# Supplementary material for: Historical museum collections and contemporary population studies implicate roads and introduced predatory bullfrogs in the decline of western pond turtles
Source: PeerJ. 2020 Jun 12;8:e9248. doi: 10.7717/peerj.9248 (PMC7295021; doi:10.7717/peerj.9248)
Supplement: Supplemental Information 10 — The distance or breakpoint where the comparison becomes significant is highlighted in yellow. [file peerj-08-9248-s010.docx]

| Distance to nearest road from centroid (m) | Number of Sites Under Distance | p-value | Average Sex Ratio of Sites Under Distance | Average Sex Ratio of Sites Over Distance |
| --- | --- | --- | --- | --- |
| 36 | 2 | 0.92966447 | 1.387390762 | 1.460828856 |
| 43 | 3 | 0.89028537 | 1.508260508 | 1.442755659 |
| 73 | 4 | 0.68415097 | 1.568695381 | 1.422272703 |
| 134 | 5 | 0.5254195 | 1.597813447 | 1.401414631 |
| 139 | 6 | 0.70282214 | 1.529923905 | 1.417640665 |
| 155 | 7 | 0.99520692 | 1.45422049 | 1.452444053 |
| 159 | 8 | 0.21163711 | 1.709942928 | 1.266302604 |
| 177 | 9 | 0.10525625 | 1.730475586 | 1.20345918 |
| 180 | 10 | 0.22303412 | 1.322368421 | 1.253843533 |
| 199 | 11 | 0.33432955 | 1.57493457 | 1.285573975 |
| 212 | 12 | 0.17503424 | 1.597099113 | 1.206240387 |
| 219 | 13 | 0.01690906 | 1.6390805 | 1.050137594 |
| 267 | 14 | 0.00927361 | 1.612777131 | 1.005998446 |
| 306 | 15 | 0.00803937 | 1.57933273 | 0.97972028 |
| 313 | 16 | 0.01708287 | 1.543124434 | 0.972960373 |
| 384 | 17 | 0.0010111 | 1.522940644 | 0.859440559 |
